# Representation of underserved communities in non-pharmacological axial spondyloarthritis trials: a scoping review protocol

**DOI:** 10.1093/rap/rkag037

**Published:** 2026-04-03

**Authors:** Alan Mathew, Yeliz Prior, Jenni Buckley, Joseph Loades, James A Prior, Jon Packham, Simone Battista

**Affiliations:** School of Health and Society, Centre for Human Movement and Rehabilitation, University of Salford, Salford, UK; School of Health and Society, Centre for Human Movement and Rehabilitation, University of Salford, Salford, UK; School of Medicine, Keele University, Keele, UK; School of Health and Society, Centre for Human Movement and Rehabilitation, University of Salford, Salford, UK; School of Medicine, Keele University, Keele, UK; Rheumatology Department, Midlands Partnership University NHS Foundation Trust, Stafford, UK; School of Medicine, Keele University, Keele, UK; Rheumatology Department, Midlands Partnership University NHS Foundation Trust, Stafford, UK; Academic Unit of Population and Lifespan Sciences, University of Nottingham, Nottingham, UK; School of Health and Society, Centre for Human Movement and Rehabilitation, University of Salford, Salford, UK

**Keywords:** protocol, scoping review, axSpA, non-pharmacological intervention, underserved communities, social determinants of health

## Abstract

**Objectives:**

To effectively address the challenges of managing axial spondyloarthritis (axSpA), improving quality of life and reducing healthcare costs, research must consider the influence of social determinants of health and broader contextual factors. These determinants play a fundamental role in shaping individuals’ experiences with axSpA non-pharmacological interventions. Underserved communities, which often face systemic barriers related to these determinants, may experience disproportionate burdens and reduced access to effective care. Despite this, there remains a limited understanding of which social determinants of health and their intersections are collected in axSpA research. This protocol aims to outline the methodology for a scoping review to map and identify how underserved communities, social determinants of health and their intersections are represented in axSpA non-pharmacological research.

**Methods:**

The Population, Concept and Context framework was used to consider the eligibility criteria for including studies, utilising an iterative process during each stage of article selection, data extraction and reporting. The scoping review will follow the guidance from the JBI. Findings will be reported following the Preferred Reporting Items for Systematic Reviews and Meta-Analyses extension for scoping reviews. The PROGRESS-Plus framework will be used to identify and categorise the collected social determinants of health.

**Conclusion:**

This review aims to provide an understanding of how social determinants of health and their intersections are collected in non-pharmacological interventions for axSpA, informing future research priorities and promoting inclusive practices. The findings will be disseminated through research publications, including peer-reviewed articles, conference abstracts and presentations and plain-language summaries for a broader audience.

## Introduction

Recent research emphasises the role of social determinants of health and intersectionality in the prevalence, diagnosis and management of different rheumatological conditions, including axial spondylarthritis (axSpA) [1]. AxSpA is an inflammatory rheumatic disease characterised by chronic and inflammatory pain, bony formations in the axial skeleton and wider systemic manifestations and comorbidities associated with inflammatory illness [2, 3]. The prevalence of axSpA ranges from 9 to 30 per 10 000 in the general population [4], with a possible underestimation due to misdiagnosis and diagnostic delays [5–7]. This condition creates a global disease burden, resulting in reduced quality of life, disability, increased healthcare costs, reduced productivity and economic impact due to work instability, presenteeism, absenteeism, early retirement and the need for social support [2, 8].

Disease outcomes are even worse in individuals from underserved communities, as preliminary findings suggest that various patient-level factors (e.g. female sex, blue-collar jobs, low education level) and country-level factors (e.g. low-income countries) are associated with poorer health outcomes [9, 10]. Previous systematic reviews have acknowledged the influence of interventions in rheumatology but also highlighted significant limitations in how inclusivity is addressed [10, 11]. Specifically, there are gaps in mapping and exploring the depth and breadth of inclusive research design, participant recruitment and reporting practices within rheumatology studies [12–14].

Underserved communities are characterised by those who have a high healthcare burden or are disproportionately underrepresented within research and practice [15]. Reasons influencing engagement include (among others) poor promotion or access to research engagement, limited financial incentives or negative financial impacts of participation, limitations in trust in either the trial or research, individual health concerns and cultural barriers [15]. This is also representative of groups that exhibit limited engagement in accessing and participating in healthcare treatment due to protected characteristics under the Equality Act [16], socio-economic factors and health status [10, 17]. The disparities created by underrepresentation are outlined by the UK government [18] and equally represented in research [19], suggesting that further exploration of factors limiting access and engagement in healthcare and research is warranted for the benefit of underserved communities. This would support addressing well-known barriers, such as resource limitations, funding constraints, healthcare infrastructure, workforce shortages, cultural and language barriers and inadequate healthcare access [20].

The management of people with axSpA is based on non-pharmacological and pharmacological interventions [21]. Core non-pharmacological interventions include physical activity, exercise and self-management education strategies [21, 22]. These strategies, which commonly target behavioural changes, can potentially exacerbate pre-existing socio-economic inequalities [22] (intervention-generated inequalities), resulting in reduced benefits for underserved populations [23]. Furthermore, resource limitations, funding constraints and the wider ripple effects of geopolitical events [20, 24] raise concerns about the accessibility and consistent application of non-pharmacological interventions in practice [25–27]. As underserved communities currently face limitations in participation and engagement in research, there are potential ripple effects in the generalisability and effectiveness of randomised controlled trials (RCTs) in healthcare practice [15]. Addressing these challenges is essential to ensuring equitable and effective patient care.

Given the strong case for broader strategies to address health, behaviour and lifestyle in axSpA [24], it is essential to examine how social determinants of health, and their intersections, are represented and reported in current research and how they may influence the accessibility and impact of non-pharmacological interventions [10, 27]. Recent literature underscores the importance of integrating contextual determinants, diversity and inclusion into research to ensure relevance and equity in care for underserved populations [11, 28, 29]. A structured framework is therefore needed to examine these factors systematically. The PROGRESS-Plus framework [30] provides a comprehensive lens for assessing social determinants of health and health inequities, encompassing dimensions such as place of residence, race/ethnicity, occupation, gender, education, socio-economic status and disability. Applying this framework enables a clearer understanding of patterns of inclusion and underrepresentation in the existing literature and supports more inclusive future research and practice [31]. This sets the foundation for implementing strategies, guidelines and processes that effectively address this limitation within axSpA research and broader contexts [32].

Therefore, a structured synthesis of the existing literature is required to identify the extent to which underserved populations are included, the extent to which relevant determinants are reported, and where gaps persist. Using the PROGRESS-Plus framework as an analytical lens, this scoping review aims to explore and map the representation of social determinants of health and their intersections within axSpA non-pharmacological intervention studies.

The specific objectives of this study will be to identify how underserved populations are represented in non-pharmacological intervention trials for axSpA; describe the extent to which social determinants of health and their intersections are reported and considered in trial design, recruitment and outcome reporting; report the characteristics of the included studies, including intervention types, settings and populations; highlight gaps in the current literature relating to the inclusion and reporting of underserved communities and inform future research priorities and trial design that support equity, reduce health disparities and improve outcomes for underserved groups with axSpA.

## Methods

This protocol outlines the methodology of a scoping review, following the guidance provided by the JBI for scoping reviews [33]. A scoping review was selected as the most appropriate design to systematically map the breadth and nature of evidence regarding the representation of social determinants of health within axSpA non-pharmacological intervention research.

### Eligibility criteria

The Population, Concept and Context (PCC) framework will be used to consider the eligibility criteria for including and excluding studies [33]. Table 1 provides a synthesis of the inclusion and exclusion criteria following the PCC framework. Studies published in languages not known by the authors (English, Italian, French, Spanish and Turkish) will be translated using a large language model (LLM).

**Table 1:** PCC framework and exclusion/inclusion criteria.

PCC framework	Inclusion criteria	Exclusion criteria	Justification for exclusion
Population	Studies on axSpA	Study not on axSpA	Outside scope of review to explore other populations than axSpA
	Adults ≥16 years of age diagnosed with axSpA	Studies relating to children, adolescents and those <16 years of age	To ensure population homogeneity and alignment with adult-focused non-pharmacological intervention guidelines in axSpA
	Studies focus on axSpA but may also discuss other conditions (relevant data will be synthesised)	Studies including axSpA alongside other conditions without disaggregated data	To ensure accurate extraction of axSpA-specific representation data
	No restrictions considered regarding gender, sex assigned at birth or other protected characteristics etc	No exclusion criteria	To ensure inclusivity and avoid introducing selection bias related to protected or demographic characteristics
Concept	RCTs, including feasibility and pilot studies where the final RCT has not yet been published	Cohort studies, case–control studies, case reports and other non-RCT primary study designs. Conference abstracts and RCT protocols will also be excluded	To ensure methodological consistency and focus on fully reported, peer-reviewed trial-based intervention evidence with complete participant and outcome data
		Literature reviews, systematic reviews and meta-analyses	Not primary data sources; used only for reference screening to identify additional eligible studies
		Stand-alone mixed methods or qualitative process evaluations embedded within trials	To maintain focus on representation and reporting within primary trial publications
	Non-pharmacological interventions, regardless of reported outcome (e.g. rehabilitation, exercise therapy, assistive technologies, digital health, self-management, patient education, community-based, behavioural or lifestyle interventions, psychological support)	Pharmacological intervention trials	To maintain focus on non-pharmacological strategies and their representation in behavioural and lifestyle research
	Studies including both pharmacological and non-pharmacological interventions where the latter is the main focus of the study	Studies where pharmacological interventions are the primary focus	To ensure alignment with the review objective examining non-pharmacological intervention research
Context	No restrictions on study setting, date, geographic location, language or social/cultural context	No exclusions based on contextual factors	To maximise inclusivity and avoid introducing geographic, cultural or contextual bias
	Social determinants of health considered during data extraction and synthesis as guided by the PROGRESS-Plus framework	No exclusion criteria	To ensure systematic assessment of representation of underserved communities across contextual factors

### Search strategy and information sources

The search strategy will use various databases aligned with axSpA research to adequately capture the depth and breadth of the available literature. Following Cochrane guidelines [34], we will include MEDLINE (via PubMed), Embase and the Cochrane Library as our primary databases. Then, considering the topic of interest, CINAHL and PsycINFO will be searched.

The search strategies were developed using a combination of MeSH terms and keywords from the PubMed database index and then converted for parity of search using the Polyglot Search Translator [35]. It is recognised that strings translated on Polyglot may include errors. Therefore, additional steps were taken to apply search terms in the same context. To ensure the rigour of the search strategy, a librarian from the University of Salford was consulted to support the development of the search strategies used. Details of search terms are included in Table 2, which outlines the terms used across the PCC framework.

**Table 2:** Search strategies.

Database	Search strategies
MEDLINE (via PubMed)	(‘Randomized Controlled Trial’ [Publication Type] OR ‘Randomised controlled trial’ [tiab] OR RCT [tiab] OR ‘Clinical Trial’ [tiab] OR ‘Controlled Clinical Trial’ [tiab]) AND (‘Axial Spondyloarthritis’ [Mesh] OR ‘Spondylarthritis’ [tiab] OR ‘AxSpA’ [tiab] OR ‘ankylosing spondylitis’ [tiab] OR ‘Axial spondyloarthritis’ [tiab] OR ‘spondyloarthropathy’ [tiab] OR ‘Non-radiographic Axial Spondyloarthritis’ [tiab] OR ‘Spondyloarthr*’ [tiab] OR ‘Ankylosing Spondyl*’ [tiab] OR ‘Spondyloarthritides, Axial’ [tiab] OR ‘Axial Spondyloarthritides’ [tiab] OR ‘Ankylosis’ [tiab] OR ‘Spondylarthritis’[tiab])
Embase (Elsevier)	(‘randomized controlled trial’/de OR ‘randomised controlled trial’:ti, ab OR rct: ti, ab OR ‘clinical trial’:ti, ab OR ‘controlled clinical trial’:ti, ab) AND (‘axial spondyloarthritis’/exp OR spondylarthritis: ti, ab OR axspa: ti, ab OR ‘ankylosing spondylitis’:ti, ab OR ‘axial spondyloarthritis’:ti, ab OR spondyloarthropathy: ti, ab OR ‘non-radiographic axial spondyloarthritis’:ti, ab OR spondyloarthr*:ti, ab OR ‘ankylosing spondyl*’:ti, ab)
Cochrane Central	(‘Randomized Controlled Trial’:pt OR ‘Randomised controlled trial’:ti, ab OR RCT: ti, ab OR ‘Clinical Trial’:ti, ab OR ‘Controlled Clinical Trial’:ti, ab) AND ([mh ‘Axial Spondyloarthritis’] OR Spondylarthritis:ti, ab OR AxSpA:ti, ab OR ‘ankylosing spondylitis’:ti, ab OR ‘Axial spondyloarthritis’:ti, ab OR spondyloarthropathy:ti, ab OR ‘Non-radiographic Axial Spondyloarthritis’:ti, ab OR Spondyloarthr*:ti, ab OR (‘Ankylosing’ NEXT Spondyl*):ti, ab OR ‘Spondyloarthritides, Axial’:ti, ab OR ‘Axial Spondyloarthritides’:ti, ab OR Ankylosis:ti, ab OR Spondylarthritis:ti, ab)
CINAHL (inc. PsychInfo)	((PT ‘Randomized Controlled Trial’) OR (TI ‘Randomised controlled trial’ OR AB ‘Randomised controlled trial’) OR (TI RCT OR AB RCT) OR (TI ‘Clinical Trial’ OR AB ‘Clinical Trial’) OR (TI ‘Controlled Clinical Trial’ OR AB ‘Controlled Clinical Trial’)) AND ((MH ‘Axial Spondyloarthritis+’) OR (TI Spondylarthritis OR AB Spondylarthritis) OR (TI AxSpA OR AB AxSpA) OR (TI ‘ankylosing spondylitis’ OR AB ‘ankylosing spondylitis’) OR (TI ‘Axial spondyloarthritis’ OR AB ‘Axial spondyloarthritis’) OR (TI spondyloarthropathy OR AB spondyloarthropathy) OR (TI ‘Non-radiographic Axial Spondyloarthritis’ OR AB ‘Non-radiographic Axial Spondyloarthritis’) OR (TI Spondyloarthr* OR AB Spondyloarthr*) OR (TI ‘Ankylosing Spondyl*’ OR AB ‘Ankylosing Spondyl*’))

### Article selection

Once the search strategy is implemented, all presented studies will be uploaded to Covidence (Covidence, Melbourne, VIC, Australia) [36]. Duplicates will automatically be removed through Covidence before starting the titles and abstracts screening. Moreover, Covidence will screen the studies using its artificial intelligence (AI) feature, which removes references that do not report RCTs, utilising the Cochrane RCT classifier. One author (A.M.) will double-check the references automatically excluded by Covidence. A.M. and J.B. will screen the titles and abstracts, with S.B. as a third reviewer in case of disagreement. Following title and abstract review, a full-text review will be completed by A.M. and J.B., with S.B. acting as a third reviewer in case of disagreement. All studies will be uploaded and accessible to all research team members, ready for data extraction. The study selection will be documented in accordance with the Preferred Reporting Items for Systematic Reviews and Meta-Analyses (PRISMA) diagram.

### Data extraction and synthesis

Data will be extracted from the selected articles by three researchers, including A.M. and J.B. Data will be charted based on an adapted JBI Standardised Data Extraction Form [33]. Data extraction is considered an iterative process based on the results retrieved. However, we expect to include the following data: authors and year; country of origin; aims/purpose; population and sample size; outcome measures; intervention type, comparator and details of these (e.g. duration of the intervention, if applicable); social determinants of health and their intersections are captured in the studies and grouped following the PROGRESS-Plus domains. To ensure rigour and consistency in mapping the available literature, the PROGRESS-Plus framework [30] will be used to capture a range of factors associated with the characteristics of underserved communities, serving as a lens for the conducting, mapping and reporting of findings. In addition to documenting individual determinants, the review will assess whether studies report intersectional analyses (e.g. interactions or subgroup analyses across multiple PROGRESS-Plus domains) or consider overlapping social identities in trial design, recruitment or outcome reporting.

Researchers will independently extract the data to form a data synthesis and discussion. No critical appraisal of the risk of bias will be performed, in line with scoping review guidelines.

## Data synthesis and planned reporting

The results will be analysed using a narrative synthesis. The analysis will summarise data from the included trials using frequency mapping of the reported characteristics within the studies. The PROGRESS-Plus framework [30] will be used for grouping of data within the study, highlighting the frequency with which they are considered within the included studies. Grouping of frequency will be considered across the following categories: place of residence’ race, ethnicity, culture, language; occupation; gender/sex; religion; education; socio-economic status; and social capital. The Plus characteristics will also be used to group data around personal characteristics associated with discrimination, features of relationships and time-dependent relationships. Using this framework will support the identification of how underserved communities have been reported to date, highlighting characteristics and groupings that have been most considered, as well as those that have been least considered. This will also inform the discussion section of the review by exploring gaps presented in the literature. The scoping review primarily intends to map the available evidence and report gaps identified within the available data. While critical appraisal is not commonly performed in alignment with the guidance on scoping reviews [33], the patterns identified could inform critical evaluation that will be included in the published review.

## Discussion

This study will examine how underserved communities are represented in clinical trials of axSpA interventions, with a particular focus on the role of social determinants and intersectionality in the current literature [1]. The study aims to identify specific populations that may face barriers to participation, engagement or access in axSpA trials. These insights could support improvements in research practice, including more inclusive recruitment strategies and comprehensive demographic reporting in future studies [11]. Specifically, this scoping review has the potential to inform the sampling of future studies to address disparities in inclusion, access and participation, contributing to efforts to reduce health inequalities recognised by both government policy and the wider research agenda [18, 19].

On a healthcare level, this review has the potential to outline implications for patient outcomes shaped by person-level factors. It may identify areas for broader changes in treatment approaches to better support diverse populations that face barriers to access [9, 37]. Moreover, the review findings may help inform clinical guidelines on non-pharmacological interventions in axSpA, enhance training for healthcare professionals and support the development of tailored interventions. Additionally, the methodology established in this review could guide future research exploring the representation of underserved communities in other health conditions.

The scoping review and its findings will be reported using the PRISMA extension for scoping reviews [38]. The studies will be disseminated through a peer-reviewed journal and shared at conferences and other relevant platforms. Implementing this approach creates the potential for significant changes in strategy development and improvements in clinical practice, policy development, clinical guidelines and educational implications, ultimately enhancing accessibility and outcomes for underserved communities [39]. In publishing this protocol, we aim to minimise potential reporting bias and enhance the study’s replicability. The full scoping review will note any deviation from the published protocol.

## Data Availability

Data are available upon reasonable request by any qualified researchers who engage in rigorous, independent scientific research, and will be provided following review and approval of a research proposal and Statistical Analysis Plan (SAP) and execution of a Data Sharing Agreement (DSA). All data relevant to the study are included in the article.
